# Transcranial Direct Current Stimulation of the Temporoparietal Junction in Autism Spectrum Disorder: Results of a Phase‐IIa Randomized, Double‐Blind, Sham‐Controlled Feasibility Study

**DOI:** 10.1002/aur.70084

**Published:** 2025-07-16

**Authors:** Christina Luckhardt, Magdalena Schütz, Andreas M. Mühlherr, Sara Boxhoorn, Christine Ecker, Hanna Mössinger, Julia Siemann, Fabienne Schlechter, Miguel Castelo‐Branco, Helena C. Pereira, Marianne Latinus, Camille Ricou, Frederique Bonnet‐Brilhault, Ricardo Salvador, Giulio Ruffini, Rafal Nowak, Michael Siniatchkin, Astrid Dempfle, Christine M. Freitag

**Affiliations:** ^1^ Department of Child and Adolescent Psychiatry, Psychosomatics and Psychotherapy, University Hospital Frankfurt Goethe University Frankfurt Germany; ^2^ Clinic of Child and Adolescent Psychiatry and Psychotherapy, Protestant Hospital Bethel EvKB Bielefeld Germany; ^3^ Coimbra Institute for Biomedical Imaging and Translational Research (CIBIT), ICNAS, Faculty of Medicine, Institute of Physiology University of Coimbra (UC) Coimbra Portugal; ^4^ UMR 1253, iBrain Université de Tours, Inserm, Centre de Pédopsychiatrie, CHRU Bretonneau Tours France; ^5^ Centre Hospitalier Universitaire de Tours (CHUT) Centre Universitaire de Pédopsychiatrie, UMR930 INSERM/Equipe Autism, CHRU Tours/Hôspital Bretonneau Tours France; ^6^ Neuroelectrics SLU Barcelona Spain; ^7^ Clinic of Child and Adolescent Psychiatry, Psychosomatics and Psychotherapy, University Hospital Aachen RWTH Aachen University Aachen Germany; ^8^ Institute of Medical Informatics and Statistics (IMIS) Kiel University Kiel Germany

## Abstract

Activation of the temporoparietal junction (TPJ) is reduced in autism spectrum disorder (ASD) during social cognitive tasks. Therefore, anodal transcranial direct current stimulation (tDCS) of the TPJ may enhance social cognitive abilities in autistic individuals. In a multicenter, randomized, sham‐controlled, double‐blind parallel‐group Phase‐IIa trial, we investigated feasibility, safety, and effect sizes of 10 sessions of anodal tDCS of the bilateral TPJ at 2 mA as an add‐on to computer‐based social cognitive training in 10‐ to 17‐year‐old youth with autism. Feasibility of recruitment was low, with only 11% of screened individuals being randomized to tDCS (*N* = 12) or sham (*N* = 12). In contrast, retention in the study, data collection, intervention adherence, and technical feasibility were mostly excellent. No serious adverse events occurred, and stimulation was well tolerated. There were no differences in the prespecified primary outcome social responsiveness between sham and tDCS immediately after the intervention (standardized estimated effect size [ES] = 0.098; 95%‐confidence interval [95% CI] −1.043;1.240), but the sham group showed a trend for better social responsiveness at the 4 week follow‐up (ES = 1.106; 95% CI −0.054; 2.270). Secondary outcomes including questionnaires and event‐related potentials showed improved compulsive behavior and quality of life by tDCS. High technical feasibility, participant retention, and safety highlight the potential of tDCS in autism and may inform future improvements in the feasibility of recruitment. The differential pattern of effect estimates indicates positive, but also potential negative effects of tDCS, which may vary due to tDCS stimulation parameters. The trial was prospectively registered in the German Clinical Trials Register (Deutsches Register für klinische Studien, DRKS, DRKS00014732).

**Summary**

- Transcranial direct current stimulation (tDCS) is a neuromodulation technique that can stimulate brain areas linked to autism.
- In a pilot study, we examined stimulation of the temporoparietal junction (TPJ) in autistic youth.
- Few technical problems, high completion rates, and good safety evaluations highlight the future potential of tDCS.
- Still, we observed a variable pattern of positive, but also possible negative effects of TPJ stimulation.
- Thus, the specific parameters of tDCS stimulation and their relation to relevant outcomes need to be differentially assessed by future studies.

## 1 Introduction

Children and youth with autism spectrum disorder (ASD) show a diverse set of strengths and abilities, but also face challenges in social communication and interaction as well as stereotyped and repetitive patterns of behavior (DSM‐5; American Psychiatric Association 2013). Group‐based social skills training is an evidence‐based intervention to support the social learning of autistic youth. Still, studies and meta‐analyses have shown only small to medium effects compared to treatment as usual (Freitag et al. 2016; Gates et al. 2017; Tachibana et al. 2017). Furthermore, social skills training does not specifically target the neural mechanisms underlying social cognition in ASD. Replicated social cognitive challenges of autistic people have been reported, among others, for theory of mind, emotion perception, and processing (Velikonja et al. 2019). The temporoparietal junction (TPJ) is a key hub of a brain network referred to as the “social brain” (Frith and Frith 2010). It is involved in most aspects of social cognition such as theory of mind (Vogeley et al. 2001) and related cognitive processes such as attention, visuo‐motor processing, speech and language, and self‐other differentiation (Donaldson et al. 2015; Geng and Vossel 2013; Williams et al. 2006). Functional magnetic resonance imaging (fMRI) studies in children and youth with ASD have replicated a differential activation of the TPJ during various social cognitive tasks compared to neurotypically developing controls (NT) (Patriquin et al. 2016), and pilot studies of fMRI neurofeedback have yielded promising results (Direito et al. 2021).

In NT, noninvasive brain stimulation of the TPJ has been shown to improve cooperation, perspective taking, and emotion attribution abilities (Donaldson et al. 2019; Santiesteban et al. 2012; Ye et al. 2015). Transcranial direct current stimulation (tDCS) is a brain‐stimulation technique applying low‐intensity electrical stimulation (0.5–2.0 mA) via anode and cathode electrodes placed on the surface of the scalp to alter resting membrane potentials in regions of the induced current flow (Stagg and Nitsche 2011). It modulates cortical excitability, resulting in changes of resting state activity (Boonstra et al. 2016) and functional connectivity of brain networks (To et al. 2018). While single sessions of tDCS often lead to small and inconsistent changes, repeated tDCS sessions lead to stronger and more consistent neurofunctional effects (Berryhill and Martin 2018). Furthermore, meta‐analytic evidence indicates that online stimulation (i.e., tDCS stimulation during performance of a cognitive task involving the stimulated area) leads to stronger improvements in cognition, especially in neurodiverse populations, such as children and youth with attention‐deficit/hyperactivity disorder (ADHD) (Dedoncker et al. 2016).

Consequently, tDCS has been considered an innovative approach to enhance social cognition in autistic individuals. To date, most randomized controlled trials on tDCS have focused on stimulating the dorsolateral prefrontal cortex (DLPFC) in autistic individuals. Studies showed reduced ASD symptoms (Amatachaya et al. 2014, 2015; Auvichayapat et al. 2022; Hadoush et al. 2020; Han et al. 2022; Qiu et al. 2021; Zemestani et al. 2022) and improved working memory by anodal DLPFC stimulation (van Steenburgh et al. 2017). Also, neural functional changes following anodal DLPFC stimulation have been reported, such as increased peak alpha frequency (Amatachaya et al. 2015) and enhanced prefrontal resting‐state functional connectivity (Han et al. 2022). To date, only a few studies have investigated parts of the so‐called “social brain” as target regions for tDCS in ASD. One small‐scale pilot study investigated the effect of a single session of anodal stimulation of the right TPJ at 2 mA with a concurrent social skills training. Behavioral results from six autistic adults indicated higher verbal fluency following tDCS stimulation compared to sham stimulation, and a trend for improved social skills (Esse Wilson et al. 2018). Another study in 16 autistic children compared anodal stimulation of the right TPJ to ventromedial prefrontal cortex (vmPFC) stimulation, and found enhanced theory of mind abilities after vmPFC stimulation only (Salehinejad et al. 2021). Taken together, tDCS stimulation is a promising approach to enhance social communication. Still, it remains unclear whether stimulation of the TPJ, a core structure within the “social brain,” supports social cognitive learning in autistic youth.

Before studying efficacy by a randomized controlled Phase‐III trial, the effect sizes of a new intervention on relevant outcome measures need to be estimated, and the feasibility of the study design as well as the intervention and its' acceptance should be shown by a Phase‐IIa study. This is essential to allow a well‐informed sample size estimation for a later confirmatory study with a feasible and acceptable study design. Besides feasibility, safety is also crucial to judge the potential translation of tDCS stimulation into routine clinical care. This is especially relevant for brain stimulation techniques, which are not yet part of the clinical routine in most healthcare systems.

The aim of the present study was therefore to conduct a pilot Phase‐IIa randomized, double‐blind, sham‐controlled, parallel‐group feasibility trial to investigate whether 10 × 20 min multichannel 2 mA anodal tDCS stimulation of the bilateral TPJ in addition to a computer‐based social cognitive training is feasible, safe, and may result in enhanced social cognitive skills in autistic children and adolescents. We studied the feasibility of recruitment, retention, intervention adherence, compliance, technical feasibility, and completeness of data collection. As the primary efficacy outcome, social responsiveness was assessed. Additionally, a comprehensive battery of secondary outcome measures, comprising data derived from questionnaires and electroencephalography‐based event‐related potentials (ERPs), were evaluated to get a full picture of behavioral effects and to study neural functional changes induced by the intervention.

## 2 Methods

The study was a multicenter, two‐arm randomized, double‐blind, parallel‐group, sham‐controlled Phase IIa pilot trial with one time‐point for screening/inclusion (T1) and three measurement time‐points (T2, baseline; T3, postintervention; T4, 4‐week follow‐up). The intervention in arm one comprised repeated anodal tDCS over the bilateral TPJ added to a computer‐based cognitive training focusing on perspective taking, intention attribution (IA), and emotion understanding. The control condition (arm two) was a blinded sham stimulation added to the cognitive training. Study sites were: (1) the Department of Child and Adolescent Psychiatry, Psychosomatics and Psychotherapy and Autism Research and Intervention Center of Excellence at the University Hospital Frankfurt, Goethe University; (2) the Department of Child and Adolescent Psychiatry, Psychosomatics and Psychotherapy at Bethel Protestant Hospital in Bielefeld, Germany; (3) the ICNAS, Centro Clínico Académico, University of Coimbra, Portugal; and (4) the Centre Hospitalier Regional Universitaire de Tours, Tours, France.

The trial protocol was prospectively reviewed and approved by all responsible national authorities and ethics committees, prospectively registered in the German Clinical Trials Register (Deutsches Register für klinische Studien [DRKS], DRKS00014732), and the study protocol has been prospectively published (Luckhardt, Schütz, et al. 2021).

### 2.1 Sample and Inclusion/Exclusion Criteria

Children and adolescents with ASD aged 10–< 18 years were eligible for the study. All participants and their caregivers gave informed consent/assent before participation in the study. The inclusion criterion was an expert clinical diagnosis of ASD according to DSM‐5 (299.00), confirmed by the Autism Diagnostic Interview‐revised or the Autism Diagnostic Observation Schedule, 2nd edition. ADOS‐2 severity scores per group are provided in Table 1.

**TABLE 1:** Descriptive data of the study sample for tDCS and sham groups.

	tDCS	Sham
*n*	12	12
Age	14.01 (2.66)	13.82 (2.57)
Female sex	1 (8.3)	2 (16.7)
Intelligence (IQ total)	103.54 (16.11)	92.08 (15.98)
Handedness		
Ambidextrous	3 (25.0)	2 (16.7)
Left‐handed	0 (0.0)	2 (16.7)
Right‐handed	9 (75.0)	8 (66.7)
ADOS 2 severity score	6.67 (1.72)	6.67 (1.78)

Exclusion criteria were an intelligence quotient (IQ) < 70 estimated based on the respective locally normed Vocabulary and Picture Completion subscales of the third edition of the Wechsler Intelligence Scale for Children or the Wechsler Adult Intelligence Scale (WISC, WASI, Wechsler 1991, 1997); birth weight < 2000 g; a neurological disorder or other somatic contraindication to tDCS; current pregnancy or hormonal contraception; history of smoking within the last 5 years; visual or hearing impairments; any comorbid mental disorder other than the following (classified according to DSM‐5): all communication disorders, ADHD, specific learning disorder with IQ ≥ 70, developmental coordination disorder, mild depressive episode, dysthymia, all anxiety disorders, all elimination disorders, gender dysphoria, oppositional defiant disorder, ASD‐related sleeping problems, and ASD‐related eating problems. Concurrent neurofeedback, stimulation therapy, or participation in other clinical trials also led to exclusion.

The following stable psychopharmacological and psychosocial interventions were allowed during study participation: any antidepressant or antipsychotic medication with a stable dosage for at least 4 weeks before baseline and until follow‐up; methylphenidate, any amphetamine preparation and melatonin with a stable dosage for at least 1 week before baseline and until follow‐up. Psychosocial interventions, such as behavioral, language or occupational therapy, were continued.

### 2.2 Randomization/Blinding

**FIGURE 1:**
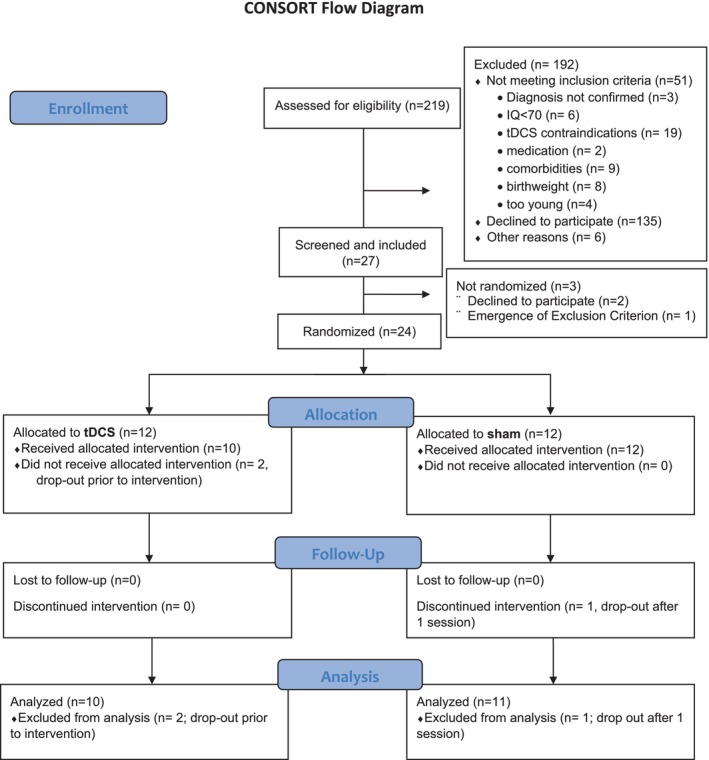
CONSORT inclusion flowchart.

Each participant was successively allocated randomly to one of the two treatment conditions (anodal tDCS stimulation or sham stimulation) by the statistician using block randomization, stratified by site and gender (Figure 1). The stimulation device Starstim 32 (Neuroelectrics) allowed for double‐blind stimulation by administering blinded stimulation protocols. Subjects, parents, and study personnel were blinded to treatment allocation for the entire duration of the trial.

### 2.3 Intervention

**FIGURE 2:**
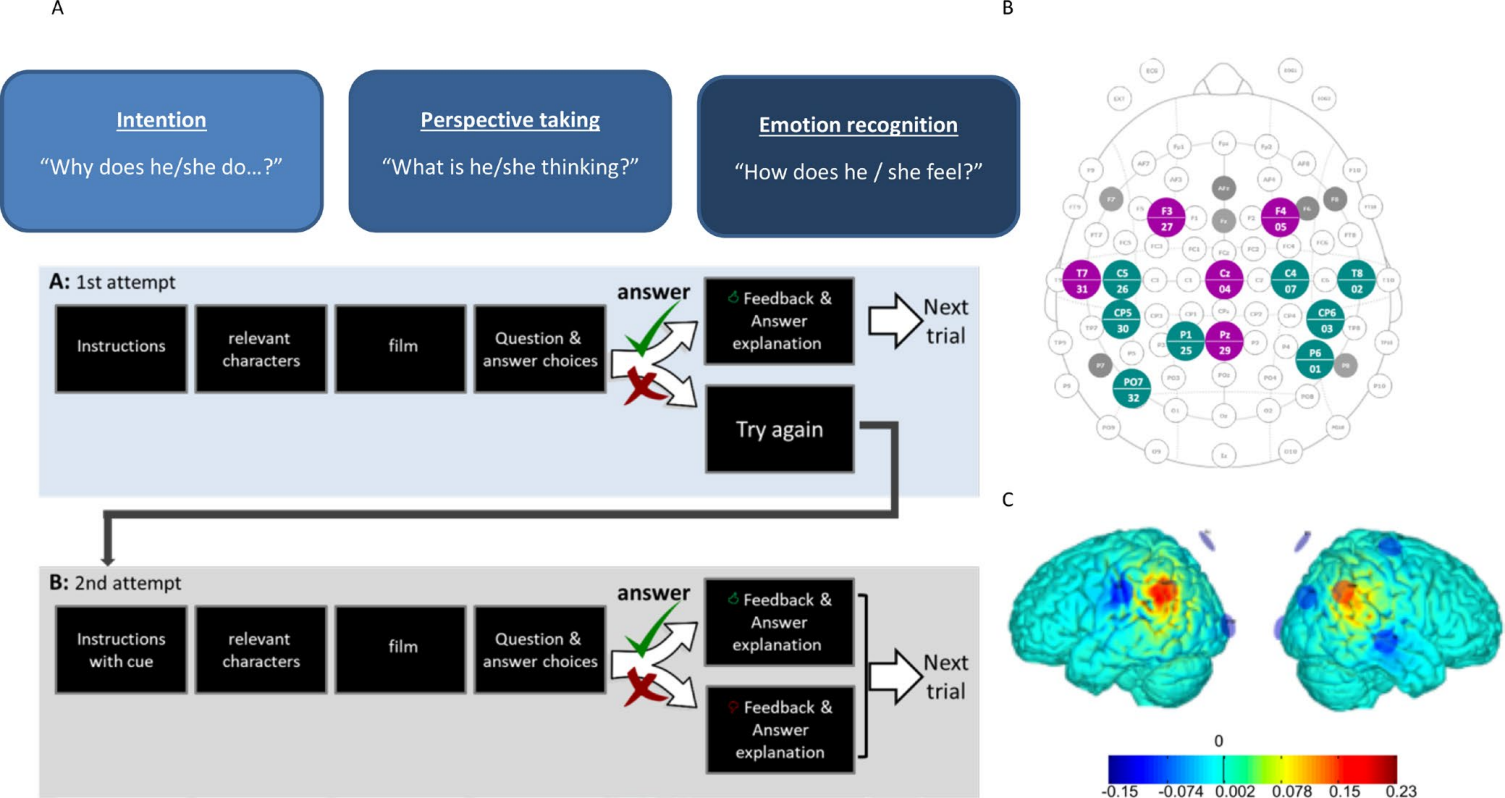
Computer‐based cognitive training and TPJ stimulation montage. (A) Schematic sequence of the socio cognitive training. (B) Electrode placement within the 10–20 system, magenta = EEG electrodes, green = stimulation electrodes. (C) Montage i: Focal montage, IM ax = 2.0 mA. Normal component of the E‐field En (V/m).

The intervention comprised 10 sessions of either tDCS or sham stimulation in addition to a computer‐based social cognitive training over a 2‐week period. The intervention group received anodal stimulation of the bilateral TPJ at 2 mA for a duration of 20 min (plus 30 s ramp‐up and 30 s ramp‐down) per session. The sham stimulation control group got the 30 s ramp‐up followed by 30 s fade‐out after 5 s, and a 30 s fade‐in and the 30 s ramp‐down at the end within a similar total duration of 21 min, but without effective stimulation. Stimulation was delivered by the Starstim 32 device (Neuroelectrics SLU, Barcelona, Spain). The TPJ was targeted by an optimized multichannel (HD‐tDCS) montage based on DTI tractography. We used 1 cm radius Ag/AgCl cylindrical electrodes with conductive gel (NG PiStim electrodes). Both the tDCS and sham groups performed a cognitive training battery targeting theory of mind and perspective‐taking abilities during the intervention sessions. The computer‐based training battery consisted of short film clips taken from the Harry Potter films dubbed in the local language, followed by questions probing the participant's understanding of the character's intentions, thoughts, or feelings, followed by feedback and additional adaptive cues for incorrect answers. An experimenter was present during the training to assist, motivate, and encourage the participant (Figure 2). All investigators were trained in the application of the cognitive training, and the use of the stimulation device and standard operating procedures (SOPs) were used to ensure high quality of intervention delivery. Study quality was ensured by extensive monitoring of all study‐related procedures and corresponding documentation carried out by independent monitors (see Luckhardt, Boxhoorn, et al. (2021) and Luckhardt, Schütz, et al. (2021) for more details).

## 3 Outcome Measures

### 3.1 Feasibility

Feasibility of recruitment was measured by documenting the number of participants contacted versus included, and by collecting reasons for nonparticipation, including the frequency of different exclusion criteria. The number of participants screened and included was also recorded.

Retention in the study was evaluated, first, by the number of dropouts. Second, for participants remaining in the study, the number of stimulation sessions attended and fully completed, the time‐period to complete the 10 sessions, and how often sessions had to be rescheduled. For each intervention session, participants' motivation was rated on a scale of 1–10, separately by the participant and the investigator. Any further issues regarding possible noncompliance were also documented by the investigator.

Technical feasibility was studied by frequency and type of technical difficulties that were reported by the investigators regarding the computer‐based training and the tDCS stimulation.

Completeness of data collection was compared based on the number of completed questionnaires and the number of EEG datasets that were available for each participant per timepoint.

### 3.2 Safety and Tolerability

Ratings and observations of safety and tolerability of tDCS stimulation were assessed at all intervention visits based on an established tDCS tolerability questionnaire (see Supporting Information). Furthermore, adverse events (AEs) and serious adverse events (SAEs) during the entire study were compared between groups. AEs and SAEs were documented in written form from randomization to the final assessment (T4).

### 3.3 Efficacy

The primary efficacy outcome measure was the effect size of the difference in parent‐rated social responsiveness (SRS‐16‐item short form [SRS‐SF] Sturm et al. 2017) between the tDCS and sham group post intervention (T3). The SRS‐SF is a short version of the Social Responsiveness Scale (SRS; Constantino and Gruber 2012), which includes items most strongly capturing ASD core symptoms. It is unidimensional in structure with a reliability > 90% and has shown a better sensitivity to change than the ADOS‐2 calibrated severity score in a recent early intervention study (Freitag et al. 2025).

Secondary outcome measures obtained at baseline (T2), post intervention (T3), and follow‐up (T4) included the Repetitive Behavior Scale‐revised (RBS‐R; Kästel et al. 2021; Lam and Aman 2007), Children's Communication Checklist‐revised (CCC‐R; Wellnitz et al. 2021), Child Behavior Checklist (CBCL; Achenbach 1991), Aberrant Behavior Checklist (ABC; Aman and Singh 1986), and all areas of health‐related quality of life according to the KIDSCREEN‐27 (parent and child). For the behavior questionnaires, higher scores indicate more symptoms; for the KIDSCREEN‐27, a higher score indicates better quality of life. Neurocognitive tasks with concurrent recording of 64‐channel EEG were implemented. This included the sequential comic strip paradigm (adapted from Brunet et al. 2000; Murdaugh et al. 2014; Schütz et al. 2021; Vistoli et al. 2015; Walter et al. 2004), which has been used to show altered activation during IA in the TPJ, right inferior frontal gyrus (rIFG) and left premotor cortex in adults with ASD (Ciaramidaro et al. 2015; Kana et al. 2014; Murdaugh et al. 2014). During the task, comic stories depicting either a story that involves physical causality (PC) or IA are presented, and the participant judges whether the presented ending matches the story in the previous picture. Each story consists of four pictures, which are each presented for 2000 ms. The first two images serve to establish context, so that at the presentation of the third image, participants are able to infer an ending to the story and predict what will happen in the fourth picture. The experiment consists of 60 trials in total: 30 trials depicting IA stories and 30 depicting PC; half of each has congruous endings and half has incongruous endings. For a detailed description, see Supporting Information and Schütz et al. (2021). Details on EEG data analysis can also be found in the Supporting Information.

## 4 Statistical Analysis

Statistical analysis was carried out in accordance with a pre‐defined statistical analysis plan. It is based on the modified intention‐to‐treat (mITT) set, including all randomized participants who provided at least one postrandomization outcome measure, irrespective of the amount of treatment received or adherence to the intervention.

Feasibility was analyzed descriptively for both study arms together. (S)AEs were tabulated by study arm. Tolerability was compared between intervention arms using a linear mixed‐effects model to account for up to 10 assessments per participant (using a random intercept per subject), adjusted for age, sex, and IQ. We calculated estimated marginal means (EMs) and standardized effect sizes for each study arm (tDCS vs. sham).

Analysis of continuous primary and secondary efficacy outcomes (SRS16, further parent‐rated questionnaires, quality of life, EEG measures) was based on the modified intention‐to‐treat set, including all randomized patients irrespective of the amount of treatment received or adherence to the intervention, but excluding those who did not start the intervention, that is, did not participate in any stimulation session. Differences between tDCS and sham conditions at T3 and T4, respectively, for continuous outcomes were estimated from a linear mixed‐effects model with covariates: study site, sex, age, IQ, and the baseline value of the respective outcome variable. All available measures of the respective outcome variable at all postrandomization time points were used in this model with an unstructured covariance matrix to model the correlation within the repeated measurements per patient over time. A time × intervention interaction term was also included in the model. From this model, both EMs for each study arm on the original scale of measurement and standardized effect sizes for the difference (tDCS—sham, thus positive effect sizes indicate higher scores in the tDCS group; with 95% confidence intervals; differences in estimates divided by the estimated population standard deviation [SD]) were calculated for T3 and T4, using the emmeans and eff_size functions of the emmeans package in R.

Analyses were performed using R version 4.2.3 (R Foundation) with packages nlme, multcomp, and emmeans, and SPSS V28.

## 5 Results

### 5.1 Feasibility

In total, *N* = 219 patients were screened for eligibility. Of these, *n* = 51 could not be included because they did not meet inclusion criteria or had contraindications for tDCS. *N* = 135 declined participation, and *n* = 6 were excluded for other reasons. *N* = 27 participants were screened and included in the study, which constitutes an inclusion rate of ~12% of the screened participants. Of the included participants, three dropped out before randomization. Consequently, *n* = 24 (11%) of the screened participants were randomized. Two more participants dropped out after baseline assessments. See Figure 1 for the CONSORT flowchart. *N* = 22 participants started treatment, and *n* = 21 participants completed the trial (see Table 1 and Table S1). One participant dropped out after one stimulation session. Thus, the drop‐out rate was 12.5% of the randomized patients. The remaining *n* = 21 participants completed all 10 planned tDCS or sham sessions. Most participants completed the intervention within the planned timeframe of 14 days (*n* = 18); the time between the first and last intervention session ranged from 9 to 18 days, with a mean duration between the first and last intervention session of 12.9 days (±2.2 days). For three participants, it was necessary to schedule an additional/repeat session. Participant motivation was rated overall positively, with a participant's mean of 6.6 (±2.4) and an investigator's mean of 7.4 (±2.0) on a scale of 1–10 (see also Table S2).

Looking at the feasibility of the social cognitive training, we analyzed the reported events during a total of *N* = 211 administered sessions. Technical difficulties (i.e., malfunctioning of the software that was used to present the cognitive training) occurred during *n* = 16 sessions (7.6%); and protocol deviations had to be reported for *n* = 16 sessions (e.g., a different training block was used than intended, or the participant finished the training earlier than the stimulation; 7.6%). There were no reports of participant noncompliance. For the stimulation, the most frequently reported (technical) difficulties were related to electrodes or high impedances (4.7%), software problems (3.3%), or device connection problems (3.3%), and discomfort due to the stimulation reported by the participant (1.9%).

Regarding the completeness of outcome measures, there was one missing report on the KIDSCREEN‐27 self‐report at baseline (for *n* = 24 randomized participants) and no missing data at T3 or T4. EEG recordings for the intention task were completed at all three time points by 20 out of 21 participants (further information in Table S3).

### 5.2 Safety and Tolerability

No SAE occurred during the course of the trial, and overall rates of reported AEs were low. The most frequently occurring AEs were headaches (*N* = 13) and discomfort (*N* = 2) after the intervention sessions. Frequencies were similar in both intervention groups (tDCS: headache *N* = 6, discomfort *N* = 1; sham: headache = 7, discomfort = 1, see also Table S4). All AEs were transient and resolved by the end of the trial. Additionally, sham and tDCS groups also showed no differences in reported sensations as assessed by the tolerability questionnaire; overall reported intensity of sensations was low. For example, overall strength of itching sensation was comparable and mild in both study arms, with EMs of 0.804 in the tDCS arm and 0.789 in the sham arm (ES = −0.023 in favor of tDCS, 95% CI −1.10 to 1.150, see also Table S5).

### 5.3 Blinding Integrity

After the last stimulation visit, of the 10 participants in the tDCS condition, 4 believed they were in the tDCS condition, 3 believed they were in the sham condition, and 3 did not answer or did not know. Of the 11 participants in the sham condition, 7 believed they were in the tDCS group, 2 believed they were in the sham condition, and 1 did not answer. Fisher's exact test revealed no indication that this rating was better than random guessing (*p* = 0.6). Blinding integrity was not formally evaluated for caregivers.

### 5.4 Primary Endpoint of Efficacy

Analysis of the primary behavior rating scale outcome, the SRS‐SF summary score at T3, showed no relevant difference between tDCS (EM 40.7, standard error [SE] 1.24) and sham group (EM 40.5, SE 1.12) with a standardized estimated effect size (ES) for the difference in the SRS‐SF summary score between tDCS and sham of 0.098 (95% CI −1.04;1.24). The variability of SRS‐SF change was high, as shown by the broad confidence intervals. This was also true for most secondary outcome measures. Thus, besides nominally significant group differences, differences with large effect sizes (> 0.80) are additionally reported below.

### 5.5 Secondary Endpoints of Efficacy—Behavior Rating Scales

A positive effect of tDCS versus sham was found on the reduction of *compulsive behavior* captured by the parent rated RBS‐R with a medium effect size (ES = −0.590, 95% CI −1.140; −0.040; tDCS EM 2.22, SE 0.41, sham EM 3.36, SE 0.345). In addition, the CBCL *social withdrawal* subscale showed a large, but not significant, effect of tDCS compared to sham at T4 (ES = ‐0.91; 95% CI −2.51; 0.69).

No further positive tDCS effects on parent‐rated behavioral symptoms of the child, but several large nonsignificant effect sizes indicating possible inferiority of tDCS versus sham were found for the following outcomes: At follow‐up (T4) the SRS‐SF raw score was lower, that is, indicating less social responsiveness difficulties, in sham (EM 37.3, SE 1.11) compared to tDCS (EM 40.4, SE 1.23), with a large effect size of ES 1.11 (95% CI −0.06; 2.27). The ABC total score (ES = 0.86; 95% CI −0.22; 1.93), *stereotypic behavior* (ES = 0.88; 95% CI −0.18; 1.94), *hyperactivity* (ES = 0.90; 95% CI −0.40; 2.19), and *inappropriate speech* (ES = 1.06; 95% CI −0.20; 2.31) subscales also showed lower scores (i.e., reduced symptoms, see also Table 2) in the sham versus the tDCS group at T3. This was maintained for *inappropriate speech* at T4 (ES = 1.24; 95% CI −0.02; 2.50). The sham group also had lower CBCL *attention problem* scores compared to the tDCS group at T3 (ES = 0.84; 95% CI −0.25; 1.93).

**TABLE 2:** Main results, means at baseline (T1), estimated marginal means and standardized effect sizes for the difference in scores between the tDCS and sham groups at post intervention (T3) and follow‐up (T4).

	Baseline (T1)		Posttreatment (T3)			Follow‐up (T4)		
	Mean (SD)		Estimated marginal mean (SE)		Standard effect estimate [95% CI]	Estimated marginal mean (SE)		Standard effect estimate [95% CI]
	tDCS	sham	tDCS	Sham		tDCS	sham	
*Symptom scales*								
SRS 16	39.90 (4.58)	41.09 (3.48)	40.7 (1.242)	40.5 (1.120)	0.098 [−1.043; 1.240]	40.4 (1.233)	37.3 (1.110)	*1.106* [*−0.054; 2.270*]
RBS‐R insistence on sameness	13.90 (8.72)	19.00 (9.31)	14.3 (2.000)	14.0 (1.730)	0.055 [−1.120; 1.230]	13.6 (2.020)	13.6 (1.740)	−0.003 [−1.200; 1.190]
RBS‐R stereotyped behavior	3.50 (3.14)	3.36 (3.75)	3.96 (1.149)	2.39 (1.075)	0.756 [−0.694; 2.210]	3.4 (1.006)	2.4 (0.937)	0.466 [−0.767; 1.700]
RBS‐R self‐injurious behavior	0.90 (1.37)	0.27 (0.65)	1.007 (0.444)	0.448 (0.417)	0.653 [−0.795; 2.100]	0.4 (0.297)	0.3 (0.273)	0.165 [−0.765; 1.090]
RBS‐R compulsive behavior	2.40 (1.78)	3.82 (2.75)	2.22 (0.414)	3.36 (0.345)	**−0.590 [−1.140; −0.040]**	2.5 (0.609)	2.8 (0.548)	−0.153 [−1.000; 0.696]
RBS‐R total	20.70 (13.08)	26.45 (12.75)	21.8 (3.08)	20.8 (2.68)	0.169 [−1.080; 1.421]	20.2 (2.900)	19.6 (2.500)	0.102 [−1.060; 1.269]
CCC‐R pragmatic‐language	22.50 (11.84)	26.82 (11.03)	25.2 (2.23)	20.8 (1.92)	0.578 [−0.158; 1.310]	24.0 (2.910)	20.4 (2.630)	0.468 [−0.550; 1.490]
CCC‐R grammatical‐semantic‐language	0.90 (1.29)	1.73 (3.04)	0.719 (0.563)	0.455 (0.485)	0.168 [−0.726; 1.060]	0.9 (0.636)	0.6 (0.562)	0.180 [−0.859; 1.220]
CCC‐R total	23.40 (12.65)	28.55 (10.96)	26.0 (2.68)	21.2 (2.30)	0.580 [−0.229; 1.390]	25.0 (3.350)	21.0 (2.990)	0.481 [−0.583; 1.540]
ABC total	41.50 (28.27)	46.91 (25.17)	43.3 (6.46)	30.3 (5.58)	*0.855* [*−0.220;1.930*]	34.1 (7.090)	30.8 (6.230)	0.219 [−0.980; 1.420]
ABC irritability	7.60 (9.61)	11.45 (10.83)	8.25 (1.54)	6.71 (1.39)	0.335 [−0.521; 1.191]	6.55 (1.690)	7.26 (1.540)	−0.155 [−1.105; 0.794]
ABC lethargy	13.10 (8.29)	13.64 (6.38)	13.19 (2.32)	9.80 (1.89)	0.691 [−0.474; 1.860]	10.3 (2.390)	9.7 (1.960)	0.118 [−1.089; 1.320]
ABC stereotypic behavior	2.70 (2.83)	3.82 (4.21)	3.24 (0.927)	1.29 (0.802)	*0.877 [−0.184; 1.940]*	2.8 (1.041)	1.8 (0.920)	0.451 [−0.760; 1.660]
ABC hyperactivity	14.10 (12.41)	12.73 (12.87)	12.64 (2.53)	8.03 (2.24)	*0.898 [−0.389; 2.190]*	8.8 (2.860)	8.1 (2.580)	0.141 [−1.343; 1.620]
ABC inappropriate speech	4.00 (3.50)	5.27 (3.29)	5.32 (0.613)	3.94 (0.553)	*1.059 [−0.195; 2.310]*	4.9 (0.613)	3.3 (0.553)	*1.240 [−0.018; 2.500]*
CBCL social withdrawal	5.70 (3.68)	5.64 (3.32)	3.98 (0.630)	4.25 (0.555)	−0.203 [−1.40; 0.997]	3.4 (0.800)	4.6 (0.727)	*−0.913 [−2.510; 0.685]*
CBCL somatic complaints	1.90 (1.52)	1.09 (1.22)	1.256 (0.296)	1.038 (0.274)	0.221 [−0.574; 1.016]	1.2 (0.320)	1.5 (0.297)	−0.342 [−1.216; 0.532]
CBCL anxious/depressed	8.20 (5.85)	7.55 (2.70)	7.3 (1.300)	6.8 (1.170)	0.171 [−1.020; 1.366]	6.1 (1.220)	6.9 (1.100)	−0.290 [−1.40; 0.819]
CBCL social problems	5.90 (2.73)	3.36 (3.38)	3.8 (0.675)	3.7 (0.557)	0.058 [−0.959; 1.070]	3.9 (0.641)	3.4 (0.521)	0.273 [−0.686; 1.230]
CBCL thought problems	3.60 (3.41)	3.27 (2.45)	2.2 (0.775)	1.9 (0.711)	0.265 [−1.170; 1.700]	1.6 (0.855)	1.7 (0.790)	−0.029 [−1.620; 1.570]
CBCL attention problems	8.40 (5.30)	6.45 (3.39)	7.4 (0.801)	5.8 (0.742)	*0.838 [−0.253; 1.930]*	6.6 (1.030)	5.6 (0.965)	0.520 [−0.924; 1.960]
CBCL rule‐breaking behavior	3.30 (3.09)	2.64 (3.17)	2.3 (0.474)	2.4 (0.424)	−0.121 [−1.390; 1.150]	2.7 (0.617)	3.0 (0.567)	−0.362 [−2.070; 1.350]
CBCL aggressive behavior	11.20 (10.81)	10.82 (9.27)	9.3 (1.180)	8.3 (1.030)	0.301 [−0.540; 1.142]	8.9 (1.28)	9.5 (1.13)	−0.147 [−1.074; 0.780]
*Health‐related quality of life*								
K27 physical well‐being self	0.32 (1.39)	−0.07 (0.52)	0.4 (0.276)	−0.01 (0.254)	0.715 [−0.658; 2.090]	0.7 (0.360)	0.5 (0.335)	0.637 [−1.210; 2.480]
K27 psychological well‐being self	1.73 (1.74)	0.81 (1.13)	2.3 (0.372)	0.5 (0.322)	**1.570 [0.697; 2.430]**	2.3 (0.392)	0.6 (0.343)	**1.510 [0.595; 2.420]**
K27 autonomy and parent relations self	0.94 (0.80)	0.58 (0.56)	1.1 (0.386)	1.2 (0.357)	−0.278 [−2.100; 1.545]	1.0 (0.308)	1.2 (0.280)	−0.456 [−1.890; 0.975]
K27 peers and social support self	0.11 (1.78)	−0.33 (1.82)	0.3 (0.675)	0.4 (0.613)	−0.093 [−1.360; 1.177]	0.1 (0.447)	1.3 (0.376)	**−0.847 [−1.640; −0.053]**
K27 school environment self	0.03 (0.96)	0.60 (1.94)	1.6 (0.664)	0.2 (0.543)	*0.917 [−0.156; 1.990]*	2.0 (0.696)	0.3 (0.578)	*1.107 [−0.043; 2.260]*
K27 physical well‐being parent	−0.06 (1.41)	0.23 (1.22)	1.2 (0.320)	0.3 (0.290)	**1.154 [0.033; 2.280]**	1.1 (0.268)	−0.1 (0.237)	**1.654 [0.727; 2.580]**
K27 psychological well‐being parent	1.46 (2.07)	1.11 (1.14)	2.4 (0.487)	1.6 (0.428)	0.495 [−0.298; 1.290]	2.3 (0.517)	1.1 (0.458)	0.750 [−0.108; 1.610]
K27 autonomy and parent relations parent	1.18 (1.16)	1.15 (0.60)	1.1 (0.262)	1.4 (0.221)	−0.463 [−1.391; 0.465]	1.3 (0.278)	1.2 (0.239)	0.127 [−0.871; 1.125]
K27 peers and social support parent	−1.12 (3.56)	−3.78 (3.27)	−0.6 (0.984)	−1.8 (0.914)	*0.839 [−1.164; 2.840]*	−0.4 (0.924)	−1.2 (0.877)	0.556 [−1.327; 2.440]
K27 school environment parent	−0.14 (1.48)	0.92 (1.78)	1.863 (0.328)	0.823 (0.313)	**0.831 [0.090; 1.570]**	1.818 (0.353)	0.679 (0.372)	**0.911 [0.043; 1.780]**

*Note*: For symptom scales positive effect sizes indicate higher scores (i.e., more symptoms) in the tDCS group; for health‐related quality of life positive effect sizes indicate higher scores (i.e., higher ratings of quality of life) in the tDCS group). Bold: CI does not include 0, italics: Standardized effect estimate > 0.8/< −0.8.

Abbreviations: ABC = aberrant behavior checklist; CBCL = child behavior checklist; CCC2‐R = children's communication checklist revised; K27 = Kidscreen 27; RBS‐R = restricted and repetitive behavior checklist revised; SRS = social responsiveness scale.

### 5.6 Secondary Endpoints of Efficacy—Quality of Life

Health‐related quality of life (KIDSCREEN‐27), related to *psychological well‐being*, was rated higher by the participants in the tDCS versus sham group at T3 (ES = 1.57; 95% CI 0.70; 2.430) and T4 (ES = 1.51; 95% CI 0.60; 2.42). Quality of *school environment* at T3 (ES = 0.92; 95% CI −0.16; 1.99) and T4 (ES = 1.11; 95% CI −0.04; 2.26) was also rated better by the tDCS group, albeit the effect was not significantly different between groups. The sham group showed a higher, but not significant, self‐rating of *peers and social support* at T4 (ES = −0.85; 95% CI −1.64; −0.05). Parents reported improved *physical well‐being* at T3 (ES = 1.16; 95% CI 0.03; 2.28) and T4 (ES = 1.65; 95% CI 0.73; 2.58) and *school environment* at T3 (ES = 0.83; 95% CI 0.09; 1.57) and T4 (ES = 0.91; 95% CI 0.04; 1.78) in the tDCS compared to the sham group. Also, non‐significant superiority of tDCS was reported in relation to *peers and social support* at T3 (ES = 0.84; 95% CI −1.16; 2.84) and T4 (ES = 0.56; 95% CI −1.33; 2.44).

### 5.7 Secondary Endpoints of Efficacy—ERPs

**FIGURE 3:**
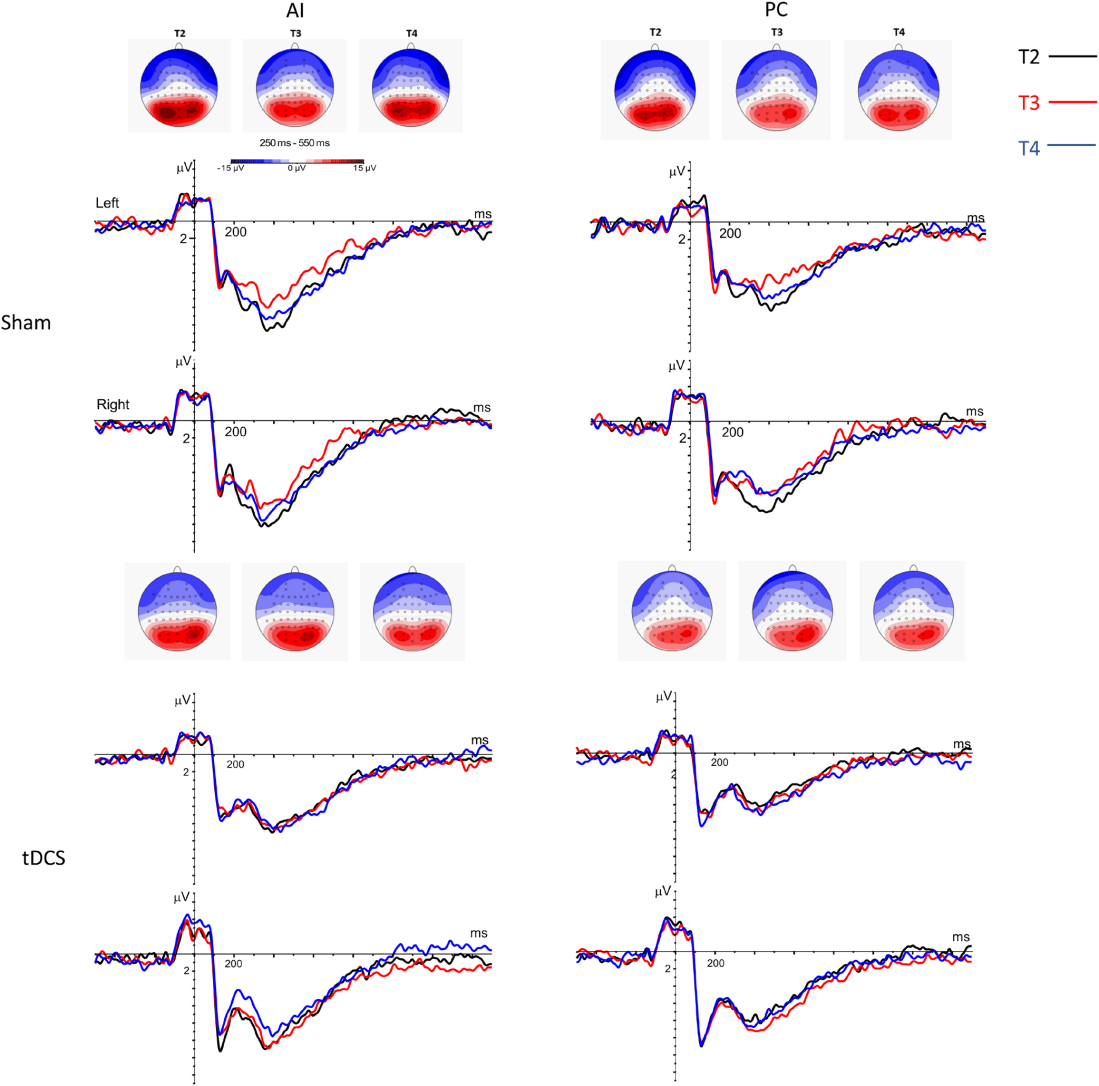
P3‐like ERP during the presentation of the third picture in the intention attribution task. Event related potentials during 250–550 ms after onset of the third image in the intention attribution task. During the task, a sequence of four pictures telling a story is presented. During the third picture, the intention of the protagonist can be inferred (intention attribution condition, AI) or physical causality (e.g., wind moving an object) can be observed (physical causality condition, PC). Topographies for the timepoints T2 (baseline), T3 (post‐intervention), and T4 (follow‐up) and corresponding grand average waveforms (black line = T2, red line = T3, blue line = T4) are shown, with sham group at the top and tDCS group at the bottom. The left column refers to the intention AI, while the right column shows the PC control condition.

ERPs and behavioral performance in the intention task were also analyzed. The confidence intervals of the ESs were high and only one effect exceeded 0.80 (see Table 3). Namely, the tDCS group showed a higher P3‐like amplitude during the IA condition in the right hemisphere (ES = 0.83; 95% CI −0.70; 2.35; Figure 3) compared to sham, which was due to a reduced P3 amplitude in the sham group at T3 compared to baseline.

**TABLE 3:** ERP and behavioral results of the intention attribution task.

	Baseline (T1) Mean (SD)		Posttreatment (T3)			Follow‐up (T4)		
			Estimated marginal mean (SE)		Standard effect estimate [95% CI]	Estimated marginal mean (SE)		Standard effect estimate [95% CI]
	tDCS	tDCS	tDCS	Sham	Standard effect estimate at T3	tDCS	Sham	Standard effect estimate at T4
AI_DPRIME	2.56 (1.49)	2.61 (1.42)	2.80 (0.719)	2.75 (0.665)	0.027 [−1.090;1.14]	2.98 (0.723)	2.67 (0.669)	0.177 [−0.949;1.300]
PC_DPRIME	2.23 (2.26)	1.74 (0.63)	3.06 (0.606)	2.80 (0.564)	0.168 [−0.856;1.192]	2.49 (0.632)	2.89 (0.591)	−0.261 [−1.339;0.817]
P3_3rd_Pic_IA_left	7.48 (4.14)	10.85 (5.57)	7.34 (1.208)	6.10 (1.113)	0.406 [−0.667;1.478]	7.29 (1.082)	7.71 (0.974)	−0.136 [−1.074;0.803]
P3_3rd_Pic_IA_right	9.01 (2.52)	10.56 (4.42)	9.21 (1.036)	7.63 (0.973)	*0.831 [−0.689;2.351]*	7.63 (0.781)	8.59 (0.694)	−0.505 [−1.581;0.571]
P3_3rd_Pic_PC_left	5.20 (4.16)	8.88 (4.85)	6.49 (1.107)	5.38 (0.905)	0.479 [−0.760;1.72]	6.83 (1.387)	6.76 (1.232)	0.028 [−1.580;1.630]
P3_3rd_Pic_PC_right	7.18 (3.66)	9.39 (4.13)	8.85 (1.120)	6.98 (0.960)	0.589 [−0.319;1.50]	8.13 (1.270)	6.97 (1.140)	0.364 [−0.701;1.430]
Diff_P3_4th_Pic_PC_left	−0.21 (3.06)	0.19 (3.50)	0.58 (1.034)	−0.62 (0.996)	0.400 [−0.568;1.370]	0.77 (1.082)	0.57 (1.045)	0.068 [−0.950;1.090]
Diff_P3_4th_Pic_PC_right	−0.51 (2.65)	0.34 (4.06)	−0.52 (1.53)	−2.23 (1.460)	0.396 [−0.581; 1.370]	0.29 (1.140)	−1.16 (1.050)	0.335 [−0.355;1.020]
Diff_P3_4th_Pic_IA_left	0.99 (3.28)	−1.21 (2.69)	−0.09 (0.903)	1.26 (0.879)	−0.589 [−1.660;0.478]	−0.64 (1.317)	1.05 (1.301)	−0.739 [−2.360;0.884]
Diff_P3_4th_Pic_IA_right	0.88 (2.86)	−1.13 (3.06)	−1.61 (1.130)	0.41 (1.100)	−0.738 [−1.840;0.364]	−1.07 (1.090)	0.30 (1.060)	−0.502 [−1.550;0.549]
Diff_Parietal_4th_Pic_ IA_left	−0.79 (1.17)	−0.46 (1.72)	−0.31 (0.775)	−0.74 (0.734)	0.221 [−0.865;1.310]	−0.88 (0.900)	−0.43 (0.866)	−0.235 [−1.524;1.050]
Diff_Parietal_4th_Pic_IA_right	−0.22 (1.97)	0.46 (1.95)	0.03 (0.979)	0.12 (0.925)	−0.042 [−1.350;1.260]	0.36 (0.827)	0.25 (0.762)	0.057 [−1.010;1.120]
Diff_Parietal_4th_Pic_ PC_left	−0.17 (1.64)	−1.69 (2.76)	0.22 (0.715)	−0.83 (0.673)	0.537 [−0.458;1.532]	−0.41 (0.570)	−0.25 (0.516)	−0.083 [−0.833;0.668]
Diff_Parietal_4th_Pic_ PC_right	0.48 (2.10)	0.01 (2.88)	0.10 (0.857)	0.37 (0.781)	−0.130 [−1.220;0.961]	−0.18 (0.903)	0.14 (0.831)	−0.154 [−1.310;1.005]
Diff_Frontal_4th_pic_ IA	0.00 (1.56)	−0.60 (2.32)	−0.84 (0.762)	−0.95 (0.700)	0.061 [−1.020;1.138]	−0.89 (0.740)	0.22 (0.675)	−0.601 [−1.640;0.441]
diff_Frontal_4th_pic_PC	−0.83 (2.04)	−0.78 (2.87)	0.46 (0.882)	1.45 (0.849)	−0.391 [−1.357;0.575]	0.403 (0.577)	0.268 (0.525)	0.264 [−0.315;0.844]

*Note:* Estimated marginal means and standardized effect sizes at postintervention (T3) and follow‐up (t4), positive effects indicate higher performance scores (DPRIME) or ERP amplitudes (μV) in the tDCS group. Italics: standardized effect estimate > 0.8/−0.8.

Abbreviations: 3rd pic = results referring to the third picture of the intention task; 4th pic = results referring to the fourth picture of the intention task; Diff = difference between correct and incorrect endings was assessed; frontal = late frontal; IA = intention attribution condition; left = left hemisphere; P3 = P3 ERP component; Parietal = late parietal ERP component; PC = physical causality condition; right = right hemisphere.

## 6 Discussion

The present study is the first randomized, double‐blind, sham‐controlled clinical trial investigating bilateral stimulation of the TPJ and its feasibility and safety, as well as establishing effect sizes for several primary and secondary outcomes, including neurophysiological measures, in children and youth with autism in detail. A high study quality was achieved by regular training of investigators and regular monitoring visits during the trial at all sites. Results on feasibility are mixed, and effect size estimates overall did not confirm expected positive effects on social responsiveness and most ASD‐associated behavior problem scales, but improvements in the child's compulsive behavior, and in parents' and children's quality of life related to well‐being and school environment by tDCS were observed.

Regarding our study design, we observed a high number of screening failures and therefore a low feasibility of recruitment of the target clinical population. Previous tDCS studies have shown a broad range of screening failure rates, ranging from none to approximately 40% (Luckhardt, Boxhoorn, et al. 2021; Prillinger et al. 2023; Qiu et al. 2021; Salehinejad et al. 2021; Zemestani et al. 2022). Our high rate of screening failures was partly due to exclusion criteria such as epilepsy and other neurological conditions, which are more common in autistic individuals than in the NT population (Lukmanji et al. 2019). Still, it may also more specifically reflect the challenges and burdens faced by families with autistic children during the Covid‐19 pandemic, which was ongoing during most of the recruitment period (Pachner and Aranyi 2021). The most frequent reason for not wanting to participate or dropping out early from the study was the number of appointments, which required coming to the clinic almost daily. Still, families and participants who committed to taking part in the study were motivated to participate, and study adherence of families, children, and intervention staff was excellent. Drop‐out during the study was only 12.5% of the randomized patients. Feasibility was also satisfactory in terms of the technical feasibility of both the computer‐based social cognitive training and the stimulation. AE rates were low, and no SAEs occurred during the study, similar to previous studies (Chhabra et al. 2020). Therefore, tDCS seems to be feasible and acceptable for parents and their autistic children motivated to participate in brain stimulation studies. For a current of 1–2 mA during tDCS stimulation, safety also is given (Buchanan et al. 2021). Quality of life rated by children and caregivers was overall better at T3 and T4 in the tDCS group. While we cannot exclude that the effect is confounded by the impact of the Covid‐19 pandemic, we can at least conclude that the tDCS stimulation has no negative effects on the perceived quality of life.

The major obstacle to the implementation of tDCS into the clinical setting was the number of stimulation sessions with daily appointments at a clinical center over 2 weeks, which was the reason for most drop‐outs, including the participant who discontinued the study after one intervention session. The time demand of the stimulation is a high barrier for most children, youth, and their families. Studies with tDCS home stimulation devices combined with home‐based cognitive training may be a way forward to improve the feasibility of tDCS stimulation as a therapeutic intervention in autism.

Regarding our primary and the secondary behavioral outcome measures, social responsiveness was similar between groups directly after the intervention, but a trend for a stronger improvement in the sham compared to the tDCS group at follow‐up was observed. Still, despite the large effect size at the four‐week follow‐up (T4), the small sample size and the high variability of outcomes in both groups do not allow definite conclusions regarding tDCS efficacy or harms. Regarding neurocognitive outcomes, we also observed no effects of the stimulation regarding task performance in the intention task. The higher amplitude in tDCS compared to sham at T3 for the P3‐like component during IA was due to a lower amplitude in the sham group at T3 compared to baseline. This reduced amplitude in the sham group may be related to the neural effects of the sociocognitive training (Luckhardt et al. 2018), in line with the SRS‐SF results at follow‐up. Still, there is only scarce research on the neural effects induced by computer‐based trainings and captured through neurophysiological outcomes. Matching our findings, a previous study found reduced P1 responses to faces in a group of autistic adults after computer‐based face expertise training (Faja et al. 2012).

Of the secondary behavioral outcomes, the RBS‐R compulsive behavior subscale was more strongly reduced in the tDCS versus sham group with a medium effect size directly after the 2‐week stimulation. The bilateral stimulation of the TPJ may have attenuated functional connectivity (Mondino et al. 2016) of neural networks involved in obsessive‐compulsive disorder (OCD) (Liu et al. 2022) and correlating with OCD symptoms in typically developing youth (Suñol et al. 2021). This hypothesis needs to be tested by adequately powered trials, also studying connectivity changes as mediators of intervention effects on OCD‐related behavior in ASD, OCD, and other mental disorders (Kästel et al. 2021).

On the other hand, there was a trend for challenging behaviors as captured by ABC subscales as well as CBCL‐derived attention problems to increase by tDCS relative to sham directly after the intervention, which were mostly not maintained at T4. Again, the small sample size, the high variability of outcomes in both groups, as well as higher inattentive symptoms, more stimulant medication, and higher IQ at baseline in the tDCS compared to the sham group do not allow definite conclusions regarding tDCS efficacy or harms in relation to challenging behaviors.

The lack of improvement of social reactivity is in contrast to findings in NT young adults, for whom meta‐analyses have reported an increase in prosocial and empathic abilities by tDCS stimulation (Bahji et al. 2021; Yuan et al. 2022). Stimulation parameters such as stimulation duration, stimulation intensity, or stimulated brain area (right TPJ, vmPFC or DLPFC) did not seem to influence results in NT young adults (Yuan et al. 2022). However, the studies included in the meta‐analysis were limited to a single session of tDCS and mainly looked at cooperative behavior during tasks such as the dictator game. One pilot study, which tested the montage used in the present study in NT adults, found no improvement in emotion recognition performance by tDCS compared to sham (Pereira et al. 2021). The two small studies in autistic individuals also did not find any improvement in social cognitive tasks with tDCS of the TPJ (Esse Wilson et al. 2018; Salehinejad et al. 2021). Both studies also applied a single stimulation session per stimulation condition/site with online performance of theory of mind tasks. Most studies implemented outcome measures directly matching the training task, that is, a theory of mind task. In contrast, we studied a distant behavior as outcome, that is, observed social responsiveness rated by parents. We chose the SRS‐SF as the primary endpoint as it captures core ASD symptoms in the social communication domain and may best reflect the impact of tDCS on daily struggles autistic people face. Given the more distant behavioral endpoint in relation to the contents of the sociocognitive training, it may not be possible to individually translate improved cognition into everyday behavior after a two‐weeks training. In line with this reasoning, several recent studies, which also implemented the SRS as an outcome in tDCS studies, have not found any effects similar to our study (Chan et al. 2023; Prillinger et al. 2023; Wang et al. 2023).

Overall, conflicting results regarding study findings in NT and autistic participants may result from differences in study design, stimulation parameters, or examined outcome measures. Still, they may also indicate group‐level differences in neural functioning between NT and autistic individuals, which by itself may lead to different outcomes of TPJ stimulation, as the underlying neural networks show differential connectivity in both groups (Cole et al. 2019). In NT, individual factors such as lateralization of TPJ activity or TPJ functional connectivity predicted different outcomes of TPJ stimulation (Yang et al. 2020). In line with this reasoning, previous studies in NT have found some indication that varying levels of autistic traits in NT may interact with the effects of anodal TPJ stimulation on neurophysiological correlates of emotional state attribution (Donaldson et al. 2018, 2019). However, the underlying mechanisms remain poorly understood and have not been studied in autistic individuals before the current study. Similar aspects may also play a role within the ASD group and may explain the high variability in outcomes within groups. Structural and functional MRI studies have found large inter‐individual differences regarding brain structure and function in autistic individuals (Ecker 2017), and brain structure predicted response to tDCS in NT (Gurr et al. 2024). These findings suggest using more individualized stimulation approaches within future trials.

Taken together, our study did not observe the hypothesized effect of tDCS improving social responsiveness by stimulation of the bilateral TPJ. Results even showed a tendency toward better social responsiveness in the sham group at follow‐up. Regarding other ASD‐related behavior, a medium effect on compulsive symptoms by tDCS was found after the stimulation, as well as positive effects on different aspects of quality of life. Even though the small sample size precludes definitive interpretation of these findings, it is interesting to consider if there are arguments for why the stimulation did not improve social responsiveness as expected. One possibility is that tDCS of the TPJ may disturb—at least when applied with the parameters of the present study—neural networks activated during processing of challenging cognitive tasks, such as our computer‐based training. A possible explanation relates to the TPJ's role in the interaction between bottom‐up and top‐down attentional control mechanisms. The TPJ has been implicated in attention re‐orienting (Corbetta et al. 2008), describing the disengagement of attention from the current attentional focus to new or unexpected stimuli, which has meta‐analytically been shown to be altered in autistic individuals (Good et al. 2024; Landry and Parker 2013). Activation of the TPJ may be advantageous to facilitate re‐orienting toward relevant or new information in simple tasks. However, with increasing task demands, it may be more advantageous to suppress this reorienting process to prevent attention from shifting away from the current task. Therefore, the dynamic activation and deactivation of the TPJ likely play an important role in the integration and modulation of bottom‐up and top‐down attentional processes (Wu et al. 2015). It seems possible that the stimulation may have prevented a dynamic adaptation of the TPJ's activation levels during the highly demanding sociocognitive training, which may explain why participants in the stimulation group did not profit from the training as much as the sham group. Following this logic, an increased activation of the TPJ may also have reduced compulsive symptoms via the facilitation of disengaging attention from compulsive thoughts. Interestingly, a relationship between increased TPJ activation and reduced compulsions has also been found in a study with patients with Tourette's syndrome (Eddy et al. 2016) and in a tDCS study of OCD (Najafi et al. 2017). Studies targeting the DLPFC, on the other hand, have found improvements in ASD symptoms (Amatachaya et al. 2014, 2015; Auvichayapat et al. 2022; Hadoush et al. 2020; Han et al. 2022; Qiu et al. 2021; Zemestani et al. 2022), which may be linked to advantageous effects of increased top‐down control during cognitive tasks.

## 7 Limitations

Like previous randomized controlled studies on tDCS, the present study can only report findings on a small sample of autistic participants. The low feasibility of recruitment prevented achieving the planned sample size of *N* = 100. Nevertheless, results give important insights into the safety and feasibility of tDCS in autistic participants. A further limitation is that within this single trial, we could not specifically evaluate parameters of the stimulation itself, such as the number of stimulation sessions and the specific stimulation montage parameters, which may have had an influence on the efficacy of the stimulation. Further research is needed to identify optimal dosage and montage parameters. The current study did also not account for neuroanatomical heterogeneity within autistic individuals. Further future study designs may consider individualizing several parameters, such as individually optimized stimulation targets, dosage, and timing, to achieve optimal effects of stimulation at the individual level (Soleimani et al. 2025).

In conclusion, the current study is the first to examine tDCS stimulation of the bilateral TPJ in such detail and to investigate its effects in terms of feasibility, safety, and efficacy. While safety, tolerability, and technical feasibility indicate tDCS as a suitable innovative intervention method for autistic individuals, the feasibility of recruitment was low, necessitating other ways of application independent of daily visits to the clinical. Effect size estimates highlight the importance of evaluating desired, as well as possible unwanted effects of tDCS intervention.

## Ethics Statement

The study protocol was prospectively submitted to the German Federal Institute for Drugs and Medical Devices (Bundestinstitut für Arzneimittel und Medizinprodukte, BfArM, Germany) and a waiver of authorization was granted. Subsequently, the trial was prospectively evaluated and approved by the ethical committee of the Faculty of Medicine at Goethe University Frankfurt (Ethik‐Kommission des Fachbereichs Medizin der Goethe‐Universität Frankfurt am Main, Germany). The waiver by BfArM and the Frankfurt ethical approval included also the German site at Bielefeld. The study protocol was also submitted to and approved by the French Drug and Health Products Safety Agency (Agence nationale de sécurité du médicament et des produits de santé, ANSM, France) and the Ethical Committee (Comité de protection des personnes, CPP, France). The study was also submitted to and approved by the National Authority of Medicines and Health Products, I.P. (Autoridade Nacional do Medicamento e Produtos de Saúde, INFARMED, Portugal) and the National Ethics Committee for Clinical Research (Comissão de Ética para a Investigação Clínica, CEIC, Portugal). The trial is referenced under EUDAMED No. CIV‐18‐01‐022765. The study was preregistered in the German Clinical Trials Register (Deutsches Register für klinische Studien [DRKS], DRKS00014732) and the study protocol has been published (Luckhardt, Schütz, et al. 2021).

## Consent

Participants were only included in the study after they and their caregivers had given written informed assent/consent.

## Conflicts of Interest

G.R. is cofounder of Neuroelectrics. R.S. and R.N. have worked at Neuroelectrics, a company that designs and produces EEG and tES solutions. C.M.F. receives royalties for books on ASD, ADHD, and MDD. She has served as a scientific advisor to the German Institute for Health Care Quality (IQWiG) and the IGES Institute during the last 3 years. She has received research funding from the State of Hessen (LOEWE), the German Ministry of Science and Education (BMBF), the German Research Association (DFG), and the European Union (EU). M.S. has received research funding from the DFG, BMBF, and EU. C.L. has received a DFG research grant. All authors carried out their work on the project within the EU‐Project STIPED. No individual compensation was received throughout the project. The other authors declare no conflicts of interest.

## Supporting information


**Data S1.**Supporting Information.

## Data Availability

Data are only available for members of the STIPED consortium in accordance with the STIPED memorandum of understanding. Other materials such as information regarding EEG tasks and the social cognitive training battery or stimulation montage are available upon request.
