# Supplementary material for: Transcranial Direct Current Stimulation of the Temporoparietal Junction in Autism Spectrum Disorder: Results of a Phase‐IIa Randomized, Double‐Blind, Sham‐Controlled Feasibility Study
Source: Autism Res. 2025 Jul 16;18(9):1861–76. doi: 10.1002/aur.70084 (PMC12442529; doi:10.1002/aur.70084)
Supplement: Supplementary file 1 — Data S1.Supporting Information. [file AUR-18-1861-s001.pdf]

Supplementary Material:

Transcranial direct current stimulation of the temporoparietal junction in autism spectrum disorder: results of a phase-IIa randomized, double-blind, sham-controlled feasibility study

Authors: Christina Luckhardt, Magdalena Schütz, Andreas M. Mühlherr, Sara Boxhoorn, Christine Ecker, Hanna Mössinger, Julia Siemann, Fabienne Schlechter, Miguel Castelo-Branco, Helena C. Pereira, Marianne Latinus, Camille Ricou, Frederique Bonnet-Brilhault, Ricardo Salvador, Giulio Ruffini, Rafal Nowak, Michael Siniatchkin, Astrid Dempfle, Christine M. Freitag

Additional information on the stimulation montage

Channel Name	Current per Electrode (mA)	Current density[1] per Electrode (mA/cm^2)	Electrode type
C4	-0,264	-0,084	NG Pistim NE029
C5	-0,581	-0,185	NG Pistim NE029
CP5	1	0,318	NG Pistim NE029
CP6	1	0,318	NG Pistim NE029
P1	-0,242	-0,0777	NG Pistim NE029
P6	-0,416	-0,132	NG Pistim NE029
PO7	-0,223	-0,071	NG Pistim NE029
T8	-0,274	-0,087	NG Pistim NE029
F3	NA	NA	NG Geltrode NE032
F4	NA	NA	NG Geltrode NE032
T7	NA	NA	NG Geltrode NE032
Cz	NA	NA	NG Geltrode NE032
Pz	NA	NA	NG Geltrode NE032

1 i.e. current / area of electrode

Additional information on outcome measures

Ratings and observations of safety and tolerability of tDCS stimulation were assessed at all intervention visits based on an established safety questionnaire [1, 2]. The questionnaire comprises 8 items regarding side effects such as itching, pain, burning, warmth, fatigue and other adverse effects. The participant is asked to rate the incidence/ intensity on a 4-point Likert-scale ("none" = 0 to "strong" = 3), to indicate when and where (if localized) the side effect occurred, as well as how disturbing it felt (on a 5-point Likert scale from "not at all" to "extremely"). Furthermore, a descriptive comparison of (S) AEs during the entire study were made between groups.

The primary efficacy estimate outcome measure was the effect size of change in parent-rated **social responsiveness (SRS-16 item short form = SRS-SF)** from baseline (T2) to postintervention (T3). The SRS-SF is a short version of the Social Responsiveness Scale (SRS) [3] which combines the raw scores of 16 items from the original SRS into a short version based on item response theory to measure autistic traits, particularly reciprocal social behavior. The SRS-SF shows high reliability (α = .96) and strong correlations with the full-length SRS (r = .98), as well as with other measures of ASD symptom severity [4].

Secondary efficacy estimate outcome measures obtained at baseline (T2), post-intervention (T3) and follow-up (T4) include the following:

– Repetitive Behavior Scale-revised (RBS-R) total score and subscales [5]. The parent-rated Repetitive Behavior Scale-Revised measures autism and obsessive compulsive disorder related repetitive behaviors. A total score and four subscales (stereotyped, self-injurious and compulsive behavior as well as persistence on sameness) are derived. Besides the subscale self-injurious behavior (α = .75), internal consistencies of the subscales are satisfying ranging from α = .81 to α = .95.

– Children's Communication Checklist-revised (CCC-R) [6]. The CCC-R is a revised version of the Children's Communication Checklist-2 [7] assessing pragmatic and semantic language abilities in children aged 4-18 years old. It contains 39 items. A total score and two subscales with high internal consistencies can be derived, i.e. CCC-R total score (α = 0.96), pragmatic-language (α = 0.96) and grammatical-semantic-language factor (α = 0.93).

– **Child Behavior Checklist 8-18 (CBCL)** [8]. The parent rated Child Behavior Checklist 6-18 is one of the most widely used valid and reliable measures in clinical research, dimensionally measuring different subscales of internalizing and externalizing behavior as well as a total score in children and youth aged 6-18 years old [9]. It contains 99 items from which a total

score, the internalizing/externalizing scores and 8 subscales (Aggressive Behavior, Anxious/Depressed, Attention Problems, Rule-Breaking Behavior, Somatic Complaints, Social Problems, Thought Problems and Social Withdrawal) are derived. Here, we studied the 8 subcales as secondary outcomes.

– **Aberrant Behavior Checklist (ABC)** [10]. The Aberrant Beavior Checklist is a parent questionnaire widely used as outcome measure in clinical trials of children and youth with autism [11]. It is a scale assessing 5 areas of challenging behaviors, such as irritability, lethargy, stereotypic behavior, hyperactivity and inappropriate speech.

– All areas of **health-related quality of life** were reported by children and separately by their parents based on the children's and parent-versions of the KIDSCREEN-27 (https://www.kidscreen.org/deutsch/frageb%C3%B6gen/kidscreen-27/). It contains 27 items covering five separate domains: physical well-being (5 items), psychological well-being (7 items), autonomy and parent relations (7 items), peers and social support (4 items), school environment (4 items). Reliability (Cronbach's alpha) is greater than 0.70 for all five dimensions. The two-week test–retest reliability ranges from 0.61 to 0.74 [12].

– **Resting state EEG** (4 min eyes open, 4 min eyes closed), **behavioral performance** (error rates and reaction times) and **neurophysiological measures** (amplitude, latency and latency variability of evoked potentials, neuronal sources of task-relevant components and oscillatory activity) were obtained by a **64-channel EEG in four tasks**. Concurrent eye tracking was optionally performed to record gaze shifts and pupil dilation.

The skills needed for the tasks are associated with TPJ functioning and are known to be impaired in ASD: (a) Intentionality was assessed in a task adapted from [13], in which comic scenes requiring the attribution of intent to a character or understanding of logical sequence of events based on physical causality are shown. (b) Visual perspective taking adapted from [14, 15] was assessed by the "dot perspective task". (c) Emotion recognition was studied by a paradigm presented with a virtual avatar whose face morphs from a neutral expression to either "happy" or "sad" expressions (https://imfar.confex.com/imfar/2016/webprogram/Paper22426.html). (d) A Posner task was used to examine abilities of attention reorienting adapted from [16, 17].

– 8-channel resting-state EEG was also recorded before and after the first, fifth and last stimulation (T2-1, T2-5 and T2-10).

Sequential Comic Strip paradigm to measure intention attribution

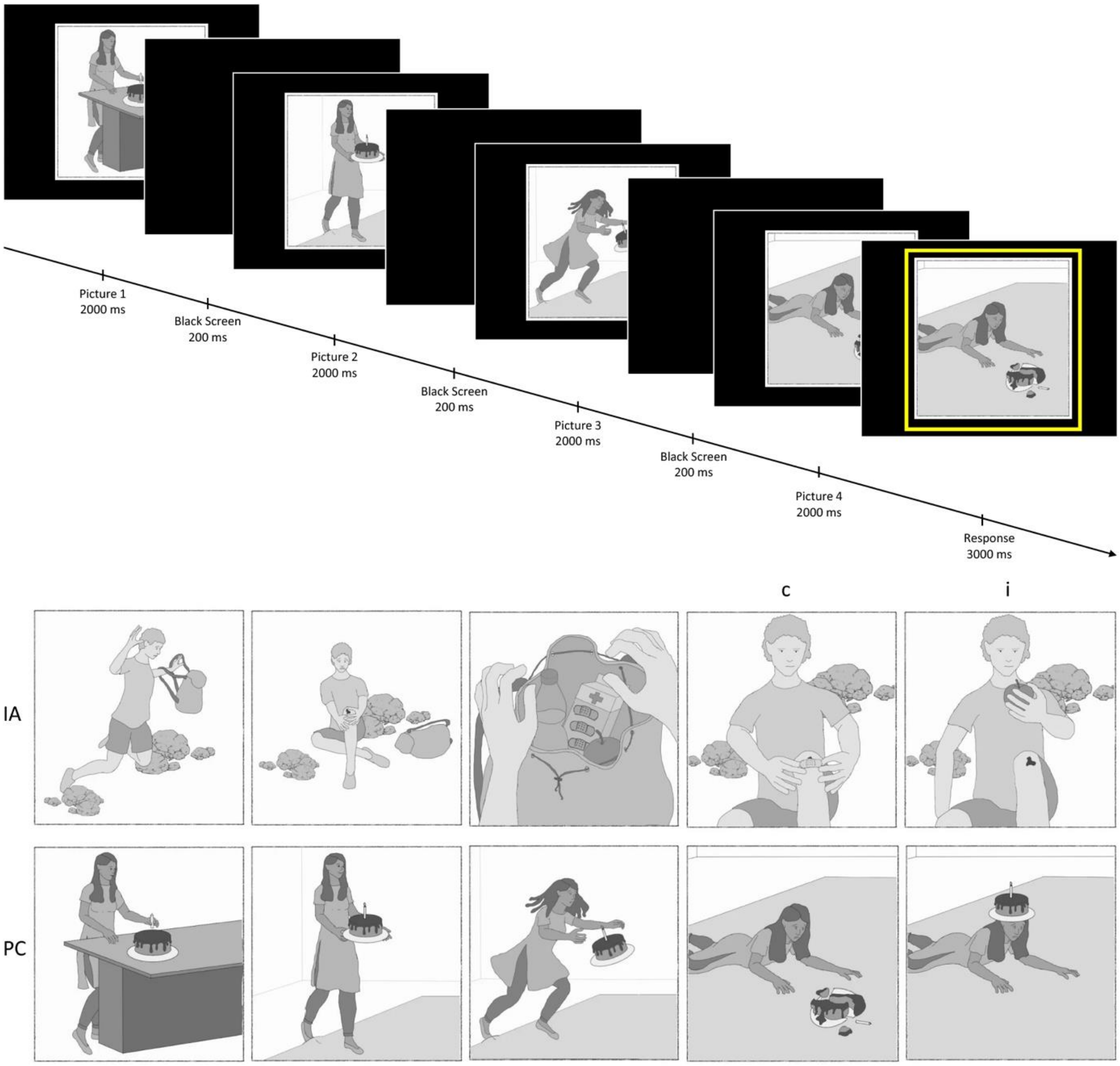

Supplementary figure 2 Intention attribution paradigm: In this paradigm four pictures constituting a story are shown sequentially. During the third image the outcome to the story becomes clear or can be predicted by the viewer. The fourth picture either shows the logical i.e. congruent ending or an incongruent one. Stories either show humans doing differnect actions (intentaion attribution / IA), or stories involving no human action, but things being affected by physical causality (PC) are presented. Participants have to indicate whether ending was congruent or not (response is delayed in order to be able to analyse what happens during the 4th picture).

EEG data analysis

EEG data was recorded at all four study sites. Analysis was carried out in Brain Vision Analyzer 2 software (Brain Products GmbH, Munich, Germany). Data was re-referenced to average reference and high-pass filtered at 1Hz (Butterworth zero phase shift 2nd order). Afterwards data was inspected for gross artefacts and ICA was performed. Components representing electro cardiac activity, blinks, eye movement or other artefacts were removed. The resulting ICA and inverse ICA matrices were then applied to data that was filtered at 0.1 Hz (Butterworth zero phase shift 2nd order). Afterwards data was segmented (-500,1000 ms) relative to the third and fourth picture, differentiating condition (PC = physical causlity, IA = intention attribution) and type of ending (congruent, incongruent) respectively. Then data was filtered at 30 Hz low-pass (Butterworth zero phase shift 2nd order), and baseline corrected (-500 ms). Semi-automatic artefact rejection was then performed on the segmented data: Finally, averages were computed for the 3rd (PC, IA) and fourth picture (PC congruent, PC incongruent, IA congruent, IA incongruent). For the 3rd picture a P3-like component was exported as a mean amplitude for 250-550 ms at two parietal-occipital clusters with electrodes PO3, PO7, P3, P5, O1 for the left & PO4, PO8, P4, P6, O2 for the right. For the 4th image, mean amplitude of the same clusters and timeframe were exported. Furthermore, mean amplitude (µV) for a frontal cluster (F1, F2, Fz) between 700 and 1200 ms after onset of the 4th image and of bilateral parietal clusters (P3, P5, CP3, CP5 for the left and P4, P6, CP4, CP6 for the right cluster) were exported to examine the effect of incongruous endings [18]. DPRIME was also computed for the IA and PC conditions as a measure of task performance.

Sample size calculation

We expected a medium effect size of Cohen's d=0.6 for the primary outcome, similar to tDCS effects in previous studies. For a power of 1-β=80% (two-sided significance level α=0.05), this leads to a sample size of 45 patients per treatment arm (G*Power3.1). Assuming 10% drop-out, the planned sample size was 100 randomized patients for each study. However, the main efficacy objective was the estimation of effect sizes, by calculating 95% confidence intervals for the difference between verum and sham in the efficacy outcome measure at T3. With the initially planned sample size of 100 patients per study, and an anticipated effect size of Cohen's d=0.6, such a 95% confidence interval was expected to have a width of 2*0.42 (standard deviation units).

Reasons for dropping out

Prior to randomization: one family felt the trial would be too time consuming, one family decided they did not wish to seek treatment for their child's autism, and one cancelled their participation because they did not want to come to the clinic during the ongoing COVID pandemic. After

baseline: one family felt the study was too time consuming, and one because the investigators discovered a previously unrecognised Tic disorder during baseline assessments.

Supplementary Table 1 Additional information on baseline characteristics of the study sample

	**tDCS**	**sham**
Included patients per recruitment site		
Bielefeld [n (%)]	3 (25.0)	2 (16.7)
Coimbra [n (%)]	0 (0.0)	1 (8.3)
Frankfurt [n (%)]	6 (50.0)	6 (50.0)
Tours [n (%)]	3 (25.0)	3 (25.0)
Detailed sample description		
Migration background of parents [n (%)]	4 (33.3)	4 (33.3)
Single parent family [n (%)]	3 (25.0)	2 (16.7)
Educational level of parents [mean (SD)]	3.17 (0.94)	2.79 (1.23)
Socioeconomic status [mean (SD)]	8.92 (1.44)	9.25 (2.93)
Pre-term delivery [n (%)]	2 (16.7)	1 (8.3)
Birth weight (g) [mean (SD)]	3215.00 (570.11)	3423.83 (590.39)
Current educational level child [n (%)]		
Primary school	3 (25.0)	4 (33.3)
Secondary school	8 (66.7)	7 (58.3)
Apprenticeship or equivalent	0 (0.0)	1 (8.3)
College	1 (8.3)	0 (0.0)
Special education	2 (16.7)	7 (58.3)
Pubertal development [n (%)]		
Prepubertal	5 (45.5)	1 (8.3)
Beginning pubertal	2 (18.2)	4 (33.3)
Midpubertal	1 (9.1)	3 (25.0)
Advanced pubertal	1 (9.1)	4 (33.3)
Postpubertal	2 (18.2)	0 (0.0)
ADI-R social interaction [mean (SD)]	17.42 (4.94)	18.92 (6.01)
ADI-R communication [mean (SD)]	12.45 (4.61)	15.00 (6.03)
ADI-R repetitive behavior [mean (SD)]	5.42 (1.88)	5.08 (2.57)
ADOS-2 social affect [mean (SD)]	7.33 (1.72)	7.33 (1.23)
ADOS-2 repetitive behavior [mean (SD)]	5.25 (2.53)	5.67 (3.17)
ADHD inattentive symptoms [mean (SD)]	3.83 (3.04)	1.92 (2.54)
ADHD hyperactive-impulsive symptoms [mean (SD)]	2.67 (2.64)	2.17 (3.10)
ADHD inattentive subtype [n (%)]	4 (33.3)	0 (0.0)
ADHD hyperactive-impulsive subtype [n (%)]	1 (8.3)	1 (8.3)
ADHD combined subtype [n (%)]	2 (16.7)	1 (8.3)
Additional current comorbid diagnoses		
Anxiety Disorder [n (%)]	3 (25.0)	2 (16.7)

Elimination Disorders [n (%)]	1 (8.3)	2 (16.7)
ODD [n (%)]	1 (8.3)	3 (25.0)
Dysthymia [n (%)]	1 (8.3)	0 (0.0)
Past Diagnoses		
Elimination Disorders [n (%)]	0 (0.0)	1 (8.3)
MDD, single episode, mild [n (%)]	0 (0.0)	1 (8.3)
Concomitant psychosocial intervention		
Speech and language therapy [n (%)]	1 (8.3)	2 (16.7)
Group based behavioral therapy [n (%)]	2 (16.7)	2 (16.7)
Individual psychotherapy	2 (16.7)	2 (16.7)
Concomitant medication		
Allergy medication [n (%)]	2 (16.7)	0 (0.0)
Homeopathic medication [n (%)]	0	3 (25.0)
Hormones [n (%)]	1 (8.3)	1 (8.3)
Melatonin [n (%)]	1 (8.3)	1 (8.3)
Non-stimulant ADHD medication [n (%)]	0 (0.0)	1 (8.3)
Nutritional supplements [n (%)]	1 (8.3)	0 (0.0)
Painkillers [n (%)]	1 (8.3)	4 (33.3)
Stimulants [n (%)]	9 (75.0)	1 (8.3)
Vaccination* [n (%)]	0 (0.0)	3 (25.0)

* Covid-19 vaccination that occured at the same time as participation in the trial.

Supplementary Table 2 Ratings of motivation during participation in the training and stimulation sessions by investigators and participants

Session	n	Mean	SD
1 participant	22	6.82	2.015
1 investigator	22	7.50	1.946
2 participant	21	6.52	2.159
2 investigator	21	7.10	2.095
3 participant	20	6.70	2.227
3 investigator	20	7.30	2.105
4 participant	21	6.33	2.331
4 investigator	21	7.71	1.848
5 participant	21	6.29	2.572
5 investigator	21	7.29	2.217
6 participant	21	6.52	2.600
6 investigator	21	7.48	1.806
7 participant	21	6.14	2.613
7 investigator	21	7.33	1.742
8 participant	21	6.38	2.598
8 investigator	21	7.24	2.119
9 participant	20	6.85	2.641
9 investigator	19	7.37	1.770
10 participant	21	6.95	2.291
10 investigator	21	7.38	2.247
rescheduled session participant	3	8.33	2.887
rescheduled session investigator	3	8.33	1.528

Supplementary Table 3 Number of completed EEG measurements per task and timepoint. *Note that the visual perspective task became optional during the conduct of the trial following feedback that measurement times were too long for participants.*

	Resting State	pos-ner task	intention task	visual perspective task	emotion recognition task
T2	24	24	24	19	21
T3	20	20	20	14	17
T4	21	18	20	13	18
all complete	19	18	20	12	16

Supplementary Table 4 Frequency of adverse events during the course of the trial, i.e. number of reports received for a given event listed by group

	**tDCS** n=12 patients	**sham** n=12 patients	Overall
Abdominal pain (upper)	0	1	1
Condition aggravated	1	0	1
Crying	0	2	2
Discomfort	1	1	2
Fatigue	2	0	2
Fever	0	1	1
Gingivitis	1	0	1
Headache	6	7	13
Ingrown nail	0	1	1
Myalgia	0	1	1
Nasal injury	0	1	1
Nasopharyngitis	1	1	1
Nausea	0	1	1
Pain in extremity	0	1	1
Testicular torsion	1	0	1
Vaccination site discomfort*	0	1	1
Vaccination site pain	0	1	1
Vomiting	0	1	1

*Note * One participant received a Covid-19 vaccination independently of the clinical trial*

Supplementary Table 5 Adverse events reported after stimulation

	tDCS (n=10)	Sham (n=11)	
	Estimated marginal mean (SE)		p-value tDCS vs sham
Itching	0.804 (0.309)	0.789 (0.264)	0.97
Pain	0.001 (0.190)	0.264 (0.162)	0.14
Burning	0.001 (0.178)	0.271 (0.153)	0.12
Warmth	0.001 (0.062)	0.043 (0.053)	0.45
Metal taste	0.003 (0.010)	0.004 (0.008)	0.90
Fatigue	0.440 (0.269)	0.692 (0.231)	0.43

Figure 1 P3-like ERP during the presentation of the 4th picture in the intention attribution task

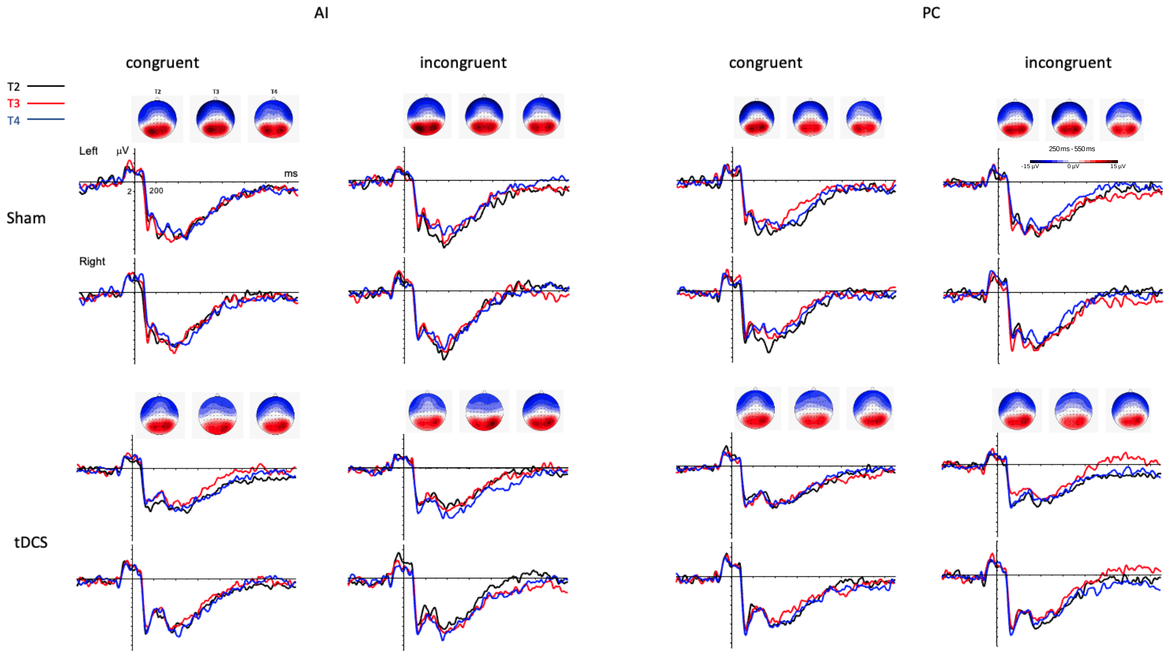

References

1. Poreisz C, Boros K, Antal A, Paulus W. Safety aspects of transcranial direct current stimulation concerning healthy subjects and patients. Brain Res. Bull. 2007;72:208–14. doi:10.1016/j.brainresbull.2007.01.004.
2. Moliadze V, Andreas S, Lyzhko E, Schmanke T, Gurashvili T, Freitag CM, Siniatchkin M. Ten minutes of 1 mA transcranial direct current stimulation was well tolerated by children and adolescents: Self-reports and resting state EEG analysis. Brain Res. Bull. 2015;119:25–33. doi:10.1016/j.brainresbull.2015.09.011.
3. Constantino JN, Gruber CP, Davis S, Hayes S, Passanante N, Przybeck T. The factor structure of autistic traits. J Child Psychol. Psychiatry. 2004;45:719–26.
4. Sturm A, Kuhfeld M, Kasari C, McCracken JT. Development and validation of an item response theory-based Social Responsiveness Scale short form. J Child Psychol. Psychiatry. 2017;58:1053–61. doi:10.1111/jcpp.12731.
5. Kästel IS, Vllasaliu L, Wellnitz S, Cholemkery H, Freitag CM, Bast N. Repetitive Behavior in Children and Adolescents: Psychometric Properties of the German Version of the Repetitive Behavior Scale-Revised. J Autism Dev Disord. 2021;51:1224–37. doi:10.1007/s10803-020-04588-z.
6. Wellnitz SAC, Kästel I, Vllasaliu L, Cholemkery H, Freitag CM, Bast N. The Revised Children's Communication Checklist-2 (CCC-R): Factor Structure and Psychometric Evaluation. Autism Res. 2021;14:759–72. doi:10.1002/aur.2467.
7. Norbury CF, Nash M, Baird G, Bishop D. Using a parental checklist to identify diagnostic groups in children with communication impairment: a validation of the Children's Communication Checklist--2. Int. J. Lang Commun. Disord. 2004;39:345–64. doi:10.1080/13682820410001654883.
8. Achenbach TM, Rescorla LA. PsycTESTS Dataset; 2001.
9. Warnick EM, Bracken MB, Kasl S. Screening Efficiency of the Child Behavior Checklist and Strengths and Difficulties Questionnaire: A Systematic Review. Child Adolesc Ment Health. 2008;13:140–7. doi:10.1111/j.1475-3588.2007.00461.x.
10. Aman MG, Singh NN, Stewart AW, Field CJ. Psychometric characteristics of the aberrant behavior checklist. Am. J. Ment. Defic. 1985;89:492–502.
11. Fung LK, Mahajan R, Nozzolillo A, Bernal P, Krasner A, Jo B, et al. Pharmacologic Treatment of Severe Irritability and Problem Behaviors in Autism: A Systematic Review and Meta-analysis. Pediatrics. 2016;137 Suppl 2:S124-35. doi:10.1542/peds.2015-2851K.
12. Ravens-Sieberer U, Auquier P, Erhart M, Gosch A, Rajmil L, Bruil J, et al. The KID-SCREEN-27 quality of life measure for children and adolescents: psychometric results from a cross-cultural survey in 13 European countries. Qual Life Res. 2007;16:1347–56. doi:10.1007/s11136-007-9240-2.
13. Vistoli D, Passerieux C, El Zein M, Clumeck C, Braun S, Brunet-Gouet E. Characterizing an ERP correlate of intentions understanding using a sequential comic strips paradigm. Soc. Neurosci. 2015;10:391–407. doi:10.1080/17470919.2014.1003272.
14. Schwarzkopf S, Schilbach L, Vogeley K, Timmermans B. "Making it explicit" makes a difference: evidence for a dissociation of spontaneous and intentional level 1 perspective taking in high-functioning autism. Cognition. 2014;131:345–54. doi:10.1016/j.cognition.2014.02.003.
15. McCleery JP, Surtees ADR, Graham KA, Richards JE, Apperly IA. The neural and cognitive time course of theory of mind. J Neurosci. 2011;31:12849–54. doi:10.1523/JNEUROSCI.1392-11.2011.
16. Sokhadze EM, Tasman A, Sokhadze GE, El-Baz AS, Casanova MF. Behavioral, Cognitive, and Motor Preparation Deficits in a Visual Cued Spatial Attention Task in Autism Spectrum Disorder. Appl. Psychophysiol. Biofeedback. 2016;41:81–92. doi:10.1007/s10484-015-9313-x.
17. Posner MI. Orienting of attention. Q J Exp Psychol. 1980;32:3–25. doi:10.1080/00335558008248231.
18. Schütz M, Boxhoorn S, Mühlherr AM, Mössinger H, Freitag CM, Luckhardt C. Intention Attribution in Children and Adolescents with Autism Spectrum Disorder: An EEG Study. J Autism Dev Disord. 2023;53:1431–43. doi:10.1007/s10803-021-05358-1.
